# COVID-19 Lockdown: Key Factors in Citizens’ Stress

**DOI:** 10.3389/fpsyg.2021.666891

**Published:** 2021-06-08

**Authors:** Susana Rodríguez, Antonio Valle, Isabel Piñeiro, Rocío González-Suárez, Fátima M. Díaz, Tania Vieites

**Affiliations:** Departamento de Psicología, Grupo de Investigación en Psicología Educativa (GIPED), University of A Coruña, A Coruña, Spain

**Keywords:** health and well-being, stress, COVID-19, context effects, survey research

## Abstract

**Background:** Confinement due to COVID-19 can have a short‐ and long-term impact on mental health (increased levels of stress and anxiety and emotional upheaval) and on people’s quality of life. Knowing what factors are behind the stress can benefit the development of strategies and resources for future situations of a similar nature. The purpose of this study is to examine the incidence of a series of sociodemographic factors, confinement conditions, and work situation on the stress reported by confined citizens.

**Method:** The sample is made up of 2008 citizens (19.9% men), the Perceived Stress Scale of 14 items (PSS-14) was used to assess the stress level of the population, as well as a sociodemographic questionnaire and different questions aimed at obtain information about the characteristics of the confinement and the employment situation. Data were collected using exponential snowball-type non-probability sampling.

**Results:** The results suggest that sociodemographic factors such as age, gender, and income level could be good predictors of confinement stress. Post-confinement work expectancy along with pre-confinement working conditions can be key to protecting the well-being of confined populations.

**Limitations:** This is a transversal study that forces us to be cautious with causal interpretations. The questionnaire was administered online, which means it excluded a good proportion of the population.

**Conclusion:** The perception of stress being higher in women than men, with the lowest stress in older people and those with higher reported incomes. Stress levels increase as populations spend more weeks in confinement and the pre-confinement work situation seems key to protecting the well-being of the population. A lower stress is observed among stable couples without children confined in residential or suburban areas. Low income or economic instability is associated with a higher rate of stress and anxiety. The results can contribute to prioritizing actions and aid by contributing to the formation of teams and the design of tools for work in the current pandemic situation.

## Introduction

In order to tackle the COVID-19 pandemic, the greatest social-healthcare challenge at the moment, unprecedented restrictions on daily life have been placed on citizens all over the world. Confinement to the home, which is what most governments chose, may have short‐ and long-term impacts on people’s mental health and quality of life. In this novel context, we look at a series of factors that may explain people’s stress response during their confinement due to COVID-19. The importance of this study lies in the opportunity to understand the factors behind confinement stress, facilitating the development and resources to deal with similar situations in the future.

### Stress and Well-Being

MERS-Cov in Korea in 2013, Serious Acute Respiratory Syndrome (SARS), and Ebola are three examples of relatively recent serious health emergencies, which had different effects on the psychological and physical health of healthcare workers and the general population. Increased stress, anxiety, emotional unrest, worry, and depressive symptoms are the most commonly reported effects in populations that have suffered confinement or a large-scale health emergency of this type (Mohammed et al., 2015; Jalloh et al., 2018; Min et al., 2018; Brooks et al., 2020; Molero et al., 2020).

Stress is conceptualized as a person’s response process when they perceive a situation or event as threatening or overwhelming due to them not having sufficient resources to deal with it (Meléndez et al., 2018). In the current situation of confinement, because of COVID-19, the perception of not controlling the environment and the sensation of being overwhelmed by events may trigger the stress process in a population (Meléndez et al., 2018). This situation demands individuals to make increased efforts and potentially compromises their health (Quick et al., 1987; Greenberg et al., 2002; Durán, 2010; Sánchez, 2013) along with the various dimensions of their well-being (Cohen and Wills, 1985; Cohen and Williamson, 1991; Cohen and Herbert, 1996; McEwen, 1998; Trujillo and González-Cabrera, 2007).

### Stress and Sociodemographic Factors

In a recent systematic review, Brooks et al. (2020) stated that sociodemographic factors may be the predictors with the greatest psychological impact on the stress of confinement. Being female has been associated with more depressive symptoms, with more anxiety, and more reported stress during the periods of confinement (Taylor et al., 2008; González-Sanguino et al., 2020; Kang et al., 2020; Pappa et al., 2020; Qiu et al., 2020; Wang et al., 2020a,b). In addition, although we have studies that finding no significant relationship between age and stress (Wang et al., 2020a), most authors suggest that people at younger (non-infant) ages would demonstrate higher rates of stress during confinement. Although those studies refer to people aged between 18 and 25 or 21–38 years old (Taylor et al., 2008; Kang et al., 2020; Ozamiz-Etxebarria et al., 2020; Qiu et al., 2020; Shanahan et al., 2020; Wang et al., 2020b), one might expect, as noted by González-Sanguino et al. (2020), age be a protective factor against the psychological impact of stress.

During a pandemic, one of the measures that governments usually employ is the cancellation of a large part of productive activity to safeguard workers and reduce transmission. This cancellation of work means workers interrupting their professional activity, often accompanied by a suspension or reduction of income. This economic instability may explain not only distress during the confinement, but also anger and anxiety once the lockdown has been lifted (Brooks et al., 2020; Lozano-Vargas, 2020; Shanahan et al., 2020; Wang et al., 2020a).

Although there are studies suggesting that educational levels do not have significant associations with indices of population stress (Hawryluck et al., 2004; Brooks et al., 2020), we have included this sociodemographic parameter in order to contribute to clarifying contradictions. Lozano-Vargas (2020) and Qiu et al. (2020) noted greater stress in individuals with higher educational qualifications (university level) based on greater awareness and understanding of the risks of the illness. However, studies, such as Wang et al. (2020a), reported that it was precisely those with the least educational qualifications who reported higher stress owing to the perception of vulnerability, lack of knowledge, and their difficulty in understanding the situation. We have also included civil or marital status as a sociodemographic variable, despite having some evidence that it cannot be significantly associated with perceived stress during the periods of confinement (Brooks et al., 2020; Lozano-Vargas, 2020; Wang et al., 2020a), as one might expect people in stable partnerships to feel more able to call on their support network of friends and family than single people, for example (Ma et al., 2020).

### Stress and Conditions of Confinement

In addition to sociodemographic characteristics, the condition in which one is confined in the home, such as whether it is with children or not, the place itself, and the length of time, may affect people’s levels of stress. Thus, apart from the stress classically associated with playing the parent–child role (Abidin, 1997; Raphael et al., 2010), one might expect that being in lockdown with children may be an additional challenge to parents who are obliged to balance full-time childcare with their own working responsibilities (Sprang and Silman, 2013; APA, 2020; Esteves et al., 2020).

Furthermore, although there is evidence that spending confinement in a densely populated city is a risk factor, affecting people’s stress (Özdin and Bayrak Özdin, 2020; Recchi et al., 2020; Tadesse et al., 2020), some authors have suggested that confinement in urban areas may even be a protective factor (Cao et al., 2020). It is possible that the population confined in urban areas would have less anxiety than those in rural areas as cities would tend to be more economically prosperous (Guessoum et al., 2020; Shigemura et al., 2020) and have better healthcare resources to cope with the disease (Cao et al., 2020).

No doubt one of the key conditions of confinement when it comes to explaining the stress response is the time that individuals have spent in confinement. We can expect that the longer the confinement, the greater the stress, and the worse the mental health (Hawryluck et al., 2004; Marjanovic et al., 2007; Reynolds et al., 2008; Brooks et al., 2020).

### Stress and Working Conditions

As we noted above, when lockdown was declared, many workers stopped going to work and had to adapt to a change in their working conditions. Remote working is a clear example, although many people had their work temporarily suspended, and in the worst cases, indefinitely suspended. Given that in the current situation, defined by severe uncertainty, working conditions during confinement may affect populations’ well-being and psychological health; in this study, we explore the extent to which they contribute to the stress response (see for example, Artazcoz et al., 2004; Brand et al., 2008; Bakioğlu et al., 2020).

Compared to those with permanent, full-time work, those most vulnerable to stress will be the unemployed or those with temporary or occasional work (Khan et al., 2002; DiGiovanni et al., 2004; Song et al., 2009; Ma et al., 2020; Mimoun et al., 2020; Shanahan et al., 2020). As we suggested above, economic security may act as a protective factor against depression, anxiety, and post-traumatic stress in the present situation (González-Sanguino et al., 2020).

## Materials and Methods

### Participants

During the period of confinement, 2008 people (19.9% men) responded voluntarily and anonymously to an online questionnaire aimed at discovering their situational stress responses and the coping strategies they were using. The respondents’ were aged between 18 and 75 years old (M_age_ = 38.30; *SD*=11.92). A total of 1745 respondents completed a Spanish version of the questionnaire, and 263 completed an English version. Although 63.2% of respondents were resident in Spain, we also received responses from various Latin American countries – 6% from Argentina, 7.5% from Ecuador, 7.6% from Mexico, among others – and from residents in the United States (11.6%).

### Instruments

We examined a series of sociodemographic factors (gender, age, civil status, educational level, and income) to determine their relationship to the stress response to confinement. In addition to recording the amount of time (in weeks) that participants had been confined, we asked about their confinement situation (with parents, parents and children, single parent and children, with a partner, or alone) and the type of residence, where they were confined (urban, rural, or suburban/residential). We also asked participants about their working conditions prior to confinement (full-time, part-time, occasional or self-employed, homemaker, pensioner, or not working/studying), the conditions of work during confinement (remote working, attending work, mixed remote and *in situ* work, temporary suspension of work, or loss of employment) and their work-related expectations for after the confinement.

To evaluate people’s levels of stress we used the 14-item Perceived Stress Scale (PSS-14) created by Cohen et al. (1983). This is a scale that has traditionally been reported to exhibit good internal and structural consistency (Cohen and Williamson, 1988; Remor, 2006; González and Landero, 2007; Campo et al., 2009; Cohen and Janicki-Deverts, 2012; Lee, 2012).

In line with theory and psychometric studies with PSS (both PSS-14 and PSS-10), in our study, it demonstrated a two-factor structure made up of elements worded positively and negatively (Taylor, 2015) which, with eigenvalues over 1, explain 52.54% of the variance. The factorial analysis we carried out for the whole sample allowed us to differentiate between control of stress (*α* = 0.83) and perceived stress (*α* = 0.85). Both chi-square from the transformation of the determinant of the correlation matrix (Bartlett’s sphericity of 0.000) and the size of the correlation coefficients (KMO = 918) indicated the suitability of the factorial structure.

### Procedure

Using non-probabilistic exponential snowball sampling, we constructed a single survey in both Spanish and English on the Microsoft Forms platform. On April 18, 2020, we published a direct link to the survey on various social networks and various other media both print and digital to publicize the request for participants in the study. The mean response time for the survey ranged between 15 and 20 min, without a time limit.

To comply with the recommendations of the Ethics Committee for Research and Teaching at the University of A Coruña and the Declaration of Helsinki (AMM, 2017), we asked participants to confirm that they were over 18. They were informed of the voluntary, anonymous, confidential nature of their participation, and they were asked to give their informed consent to participate.

Once we had achieved a sufficiently large sample, and given the beginning of loosening lockdown measures in some countries, we closed access to the survey on May 19, 2020 and began data analysis using the SPSS statistical package.

### Data Analysis

Predictor equations for stress during COVID-19 confinement were produced using logistical regression, following the forward stepwise regression procedure based on the Wald statistic. Three logistical regressions were performed using sociodemographic variables, confinement conditions, and work-related variables as predictors. The three cases included perceived stress (No = 0 or Yes = 1) as the criterion variable, referring to the mean in the Perceived Stress factor of the PSS-14 in the sample. The fit of the models was assessed using Nagelkerke’s *R*^2^ (Nagelkerke, 1991) and the percentage of correctly classified cases.

## Results

### Sociodemographic Variables and Perceived Stress During Confinement

Considering gender, civil status, the level of education, and income levels, we produced a *sociodemographic model* in order to make estimations about the mean level of perceived stress during confinement (no stress = 0/stress = 1). The categorical variables in the regression equation were coded as described in Table 1.

**Table 1:** Frequencies and parameter coding (1) for the sociodemographic variables included in the regression equation.

		Frequency	Parameter coding^*^		
			(1)	(2)	(3)
Gender	Male	400	1		
	Female	1,607	0		
Civil status	Married or stable partnership	1,225	1	0	0
	Separated or divorced	134	0	1	0
	Single	619	0	0	1
	Widowed	29	0	0	0

* Presence/absence of category.

The final explanatory model of stress would allow the correct classification of 60.3% of the sample (*χ*^2^ = 128.964; *p*=0.000), with better sensitivity in estimating above-average stress (63.3%) than below-average stress (57.1%; see Table 2).

**Table 2:** Omnibus tests on the sociodemographic model coefficients.

		*χ*^2^	*df*	Sig.
Step 1	Step	83.305	1	0.000
	Block	83.305	1	0.000
	Model	83.305	1	0.000
Step 2	Step	38.189	1	0.000
	Block	121.493	2	0.000
	Model	121.493	2	0.000
Step 3	Step	7.470	1	0.006
	Block	128.964	3	0.000
	Model	128.964	3	0.000

The analysis of the final step suggested the inclusion of three sociodemographic models: gender, age, and income, with the remaining sociodemographic variables included initially – educational level and civil status – not providing better information for the prediction of stress in confinement.

The stepwise regression process showed that *age* was the sociodemographic variable that most explained the perception of stress in confinement (*W* = 57.419; *p* < 0.001), with *gender* making a reasonable contribution to this perception (*W* = 36.494; *p* < 0.001). *Income level* would also explain perceived stress during confinement, with lower explanatory power (W = 7.398; *p* < 0.01; see Table 3).

**Table 3:** Variables in the equation.

	B	SE	Wald	*df*	*p*	*Exp*(*B*)
Step 1^a^						
Age	−0.200	0.022	80.362	1	0.000	0.818
Constant	0.784	0.099	63.250	1	0.000	2.190
Step 2^b^						
Gender (1)	−0.721	0.119	36.967	1	0.000	0.486
Age	−0.201	0.023	79.120	1	0.000	0.818
Constant	0.926	0.103	81.551	1	0.000	2.525
Step 3^c^						
Income level	−0.124	0.046	7.398	1	0.007	0.883
Gender (1)	−0.717	0.119	36.494	1	0.000	0.488
Age	−0.181	0.024	57.419	1	0.000	0.835
Constant	1.303	0.174	56.065	1	0.000	3.682

a Variable added in step 1: age.

b Variable added in step 2: gender.

c Variable added in step 3: income level.

Although the percentage of variance explained was low (Nagelkerke’s R^2^ =0.083), looking at the parameter coding, we can interpret it as the perception of stress being higher in women than men, with the lowest stress in older people and those with higher reported incomes (see Table 3).

### Confinement Conditions and Perceived Stress

In order to estimate the mean level of stress perceived by the population during confinement (no stress = 0/stress = 1), we included the length of time confined, the situation in the home, and the type of residence in the *confinement conditions model*. The categorical variables in this regression equation were coded as shown in Table 4.

**Table 4:** Frequencies and parameter coding (1) for the categorical variables related to the confinement conditions included in the regression equation.

		Frequency	Parameter coding^*^			
			(1)	(2)	(3)	(4)
Confined with…	Alone/without children	317	1	0	0	0
	With partner/without children	354	0	1	0	0
	Single parent/with children	244	0	0	1	0
	Two parents/with children	854	0	0	0	1
	With parents	117	0	0	0	0
Type of residence	Rural	335	1	0		
	Residential/Suburban	366	0	1		
	Urban	1,185	0	0		

* *Presence/absence of category*.

The final explanatory model for the perceived level of stress allowed the correct classification of 55% of the sample (*χ*^2^ = 30.662; *p* = 0.000; see Table 5) with better sensibility when estimating above-average stress (58.8%) than below-average stress (51.3%).

**Table 5:** Omnibus tests for the confinement conditions model coefficients.

		*χ*^2^	*df*	Sig.
Step 1	Step	8.290	1	0.004
	Block	8.290	1	0.004
	Model	8.290	1	0.004
Step 2	Step	10.040	2	0.007
	Block	18.329	3	0.000
	Model	18.329	3	0.000
Step 3	Step	12.333	4	0.015
	Block	30.662	7	0.000
	Model	30.662	7	0.000

The stepwise regression procedure showed that the *length of confinement* (*W* = 8.815; *p* < 0.01), the *type of residence* (*W* = 10.017; *p* < 0.01), and the *confinement situation* (*Confined with*; *W* = 12,209; *p* < 0.05) contributed to explaining the perception of stress in the population (see Table 6).

**Table 6:** Variables in the equation.

			E.T.	Wald	*df*	Sig.	*Exp* (*B*)
Step 1^a^	Weeks confined	0.174	0.061	8.246	1	0.004	1.190
	Constant	−0.466	0.168	7.696	1	0.006	0.627
Step 2^b^	Weeks confined	0.176	0.061	8.351	1	0.004	1.192
	Type of residence			9.949	2	0.007	
	Type of residence (1)	−0.033	0.124	0.071	1	0.790	0.967
	Type of residence (2)	−0.378	0.121	9.746	1	0.002	0.686
	Constant	−0.392	0.173	5.133	1	0.023	0.675
Step 3^c^	Weeks confined	0.182	0.061	8.815	1	0.003	1.200
	Confined with			12.209	4	0.016	
	Confined with (1)	−0.375	0.220	2.910	1	0.088	0.687
	Confined with (2)	−0.634	0.219	8.409	1	0.004	0.531
	Confined with (3)	−0.336	0.229	2.157	1	0.142	0.715
	Confined with (4)	−0.253	0.201	1.580	1	0.209	0.776
	Type of residence			10.017	2	0.007	
	Type of residence (1)	−0.078	0.125	0.389	1	0.533	0.925
	Type of residence (2)	−0.385	0.122	10.009	1	0.002	0.680
	Constant	−0.059	0.247	0.057	1	0.811	0.943

a Variables added in step 1: weeks confined.

b Variables added in step 2: type of residence.

c Variables added in step 3: confined with.

Although the variance explained was low (Nagelkerke’s *R*^2^ = 0.022), perceived stress would be lower in those confined with a partner, without children, and in residential or suburban areas (see Table 6). As expected, perceived stress would tend to be higher the longer the confinement (see Table 6).

### Work Situation and Stress of Confinement

Considering people’s work situation before confinement, during confinement, and their work-related expectations for after confinement, we produced a logistical regression model to assess the mean level of perceived stress (no stress = 0/stress = 1). The categorical variables for this *work situation model* were coded as shown in Table 7.

**Table 7:** Frequencies and parameter (1) coding for the categorical variables related to the work situation included in the regression equation.

		Frequency	Parameter coding^*^			
			(1)	(2)	(3)	(4)
Usual work situation	Full-time	1,059	1	0	0	0
	Part-time/occasional/self-employed	336	0	1	0	0
	Homemaker	18	0	0	1	0
	Pensioner/retired	10	0	0	0	1
	Not working/studying	107	0	0	0	0
Work situation during confinement	Remote working and attending work	114	1	0	0	0
	Remote working	687	0	1	0	0
	Attending work	306	0	0	1	0
	Temporary suspension	334	0	0	0	1
	Lost job	89	0	0	0	0

* *Presence/absence of category.*

The final explanatory model would allow the correct classification of 58.1% of the sample (*χ*^2^ = 43.602; *p* = 0.000) with better sensitivity when assessing below-average stress (66.1%; see Table 8).

**Table 8:** Omnibus tests for the workplace situation model coefficients.

		*χ*^2^	*df*	Sig.
Step 1	Step	31.820	1	0.000
	Block	31.820	1	0.000
	Model	31.820	1	0.000
Step 2	Step	11.782	4	0.019
	Block	43.602	5	0.000
	Model	43.602	5	0.000

Analysis of the final step for the explanation of perceived stress suggests that the post-confinement work-related expectations (*W* = 24.6060; *p* < 0.001) and people’s normal pre-confinement work situations (*W* = 11.593; *p* < 0.05) would contribute to their perceptions of stress. From these parameters, the work situation during confinement appears not to provide more information for the prediction of mean stress in the confined population.

Although the percentage of variance explained was low (Nagelkerke’s *R*^2^ = 0.037), looking at the parameter coding, we can interpret that perceived stress is higher the worse the post-confinement work-related expectations, and that the stress reported by those with full-time jobs is lower than the stress reported by those in other circumstances of work (see Table 9).

**Table 9:** Variables in the equation.

		B	E.T.	Wald	*df*	Sig.	*Exp*(*B*)
Step 1^a^	Work expectations	−0.533	0.095	31.130	1	0.000	0.587
	Constant	0.838	0.164	25.960	1	0.000	2.311
Step 2^b^	Work expectations	−0.480	0.097	24.660	1	0.000	0.619
	Usual work			11.593	4	0.021	
	Usual work (1)	−0.612	0.214	8.207	1	0.004	0.542
	Usual work (2)	−0.347	0.231	2.251	1	0.134	0.707
	Usual work (3)	−0.103	0.531	0.038	1	0.846	0.902
	Usual work (4)	−0.489	0.669	0.535	1	0.465	0.613
	Constant	1.257	0.248	25.616	1	0.000	3.515

a Variables added in step 1: work expectations.

b Variables added in step 2: usual work.

## Discussion

The results of this study may contribute to the recognition of factors underlying the stress of confined populations and may potentially inform possible future decisions in similar situations.

In line with the work by Brooks et al. (2020), the sociodemographic models demonstrate better explanatory power for stress in confinement than other models. Specifically, age (Taylor et al., 2008; Kang et al., 2020; Ozamiz-Etxebarria et al., 2020; Qiu et al., 2020; Shanahan et al., 2020; Wang et al., 2020b) and gender (Taylor et al., 2008; González-Sanguino et al., 2020; Kang et al., 2020; Pappa et al., 2020; Qiu et al., 2020; Wang et al., 2020a,b) seem to be the factors that best explain the perception of stress in confinement. The results also link low income and financial instability with the higher rates of stress and anxiety (Brooks et al., 2020; González-Sanguino et al., 2020; Lozano-Vargas, 2020; Shanahan et al., 2020; Wang et al., 2020a). The perception of confinement stress is higher among women than among men and decreases with age and with higher reported income.

Once age, gender, and income are considered, neither the level of education nor civil status provide better information in the explanation of stress in confinement (Hawryluck et al., 2004; Brooks et al., 2020; Lozano-Vargas, 2020; Wang et al., 2020a). Although one might expect that the level of education might make it easier for someone to properly interpret the information we are exposed to throughout confinement, the fact is that, in this study, we did not find differences in perceived stress according to this variable. It may be useful to explore potential differences in coping methods and in control of the stress response.

Although with more variance to explain than in the sociodemographic model, people’s expectations about post-confinement work together with their pre-confinement work situation may to a large extent be estimators of emotional well-being – low perceived stress – in confined populations. In this regard, and as we hypothesized, perceived stress seems to be higher as people believe that their work situations will worsen post-confinement. Positive expectations were a protective factor against stress in confinement, enhancing the well-being of confined populations by strengthening self-efficacy and reducing behaviors associated with frustration and pessimism (Dubow et al., 2001; Besser and Shackelford, 2007; Bakioğlu et al., 2020; Molero et al., 2020; Salas-Nicás et al., 2020).

In line with results from previous studies, which reported part-time workers reporting more stress than full-time workers (DiGiovanni et al., 2004; Mimoun et al., 2020), those with full-time jobs may demonstrate less perceive stress during the periods of confinement. Those who work part-time, occasionally, or are self-employed would tend to have greater difficulties dealing with confinement because of the instability of the job market and/or lower incomes. Once the less negative work-related expectations associated with more stable pre-confinement work situations are considered, the work situation during confinement does not provide more information to the prediction of people’s stress (Ma et al., 2020; Mimoun et al., 2020; Shanahan et al., 2020).

Although the variance explained by the model using confinement conditions in the home is low, our results suggest an increase in the rates of stress according to the length of confinement (Hawryluck et al., 2004; Marjanovic et al., 2007; Reynolds et al., 2008; Brooks et al., 2020; Taylor et al., 2020) and indicate a profile of reduced stress in stable couples without children (Sprang and Silman, 2013; APA, 2020; Esteves et al., 2020; Ma et al., 2020) confined in residential or suburban areas (Özdin and Bayrak Özdin, 2020; Recchi et al., 2020; Tadesse et al., 2020).

The difficulty of couples with children in accessing their support networks during the current confinement due to COVID-19 (Ma et al., 2020) may contribute to the levels of reported stress, and suburban or residential areas may combine the best qualities of urban and rural areas, contributing to a smaller stress response to confinement (Özdin and Bayrak Özdin, 2020; Recchi et al., 2020; Tadesse et al., 2020). associated with confinement in residential areas would not have the negative characteristics associated with confinement in rural areas, as residential areas are relatively closer to more urban areas, they are associated with greater economic prosperity, with better connections to public services, and better healthcare conditions in the fight against the pandemic (Cao et al., 2020; Guessoum et al., 2020; Shigemura et al., 2020).

## Data Availability Statement

The datasets presented in this study can be found in online repositories. The names of the repository/repositories and accession number(s) can be found at doi: 10.5281/zenodo.4020364.

## Ethics Statement

The studies involving human participants were reviewed and approved by the Ethics Committee of University of A Coruña. The patients/participants provided their written informed consent to participate in this study.

## Author Contributions

AV and IP conceived the presented idea. SR developed the theory and performed the computations. AV verified the analytical methods and supervised the project. IP encouraged TV and RG-S to investigate stress in COVID-19 and supervised the findings of this work. TV wrote the manuscript with support from RG-S, FD, and IP who fabricated the first sample. SR and AV helped to supervise the project and conceived the original idea. VT, RG-S, and FD developed the theoretical formalism. SR and IP performed the analytic calculations and the numerical simulations. All authors provided critical feedback, discussed the result, and also carried out the experiment. All authors contributed to the article and approved the submitted version.

### Conflict of Interest

The authors declare that the research was conducted in the absence of any commercial or financial relationships that could be construed as a potential conflict of interest.
